# Sir Arbuthnot Lane on Venereal Disease

**Published:** 1920-01-10

**Authors:** 


### Sir Arbuthnot Lane on Venereal Disease.

On Wednesday, December 17, Sir Arbuthnot Lane, Bart., C.B., delivered an address on the prevention of venereal disease by immediate self-disinfection of men, and treated this subject in a manner consistent with his broad-minded views on most surgical matters, following no written dogma, but rather fearlessly advancing his own particular opinions. After defining at some length the objects of the society he represented, he continued: "It is most important that the younger members of the community should be instructed in the details of their physiology, and that knowledge of their sexual functions should no longer be regarded as improper and indelicate. They realised, unfortunately, that in spite of the large amount of moral propaganda which had been spread broadcast among the community, it was an indisputable fact that irregular intercourse had greatly increased and that the average moral code of young women had altered very materially for the worse. It was obvious that in the vast majority of cases chastity was the only way by which venereal diseases could be avoided; yet they recognised that the nature of mankind was such that irregular intercourse would continue to take place in the future, as it had done in the past, and that means must be adopted to reduce infection to a minimum in order that the community should suffer as little as possible, and that these diseases should be eliminated."

Sir Arbuthnot emphasised the fact that if they wished to raise the standard of medical education in these matters they must also educate the general public at the same time, so that the masses, being well versed in hygiene, would insist upon more skilled treatment. Up to the present the society had limited itself to the distribution of printed leaflets, but in the near future they hoped to have an improved method of spreading knowledge broadcast among the lay public, and thus to reduce and finally exterminate these terrible scourges.

